# Vitamin D Insufficiency Predicts Susceptibility of Parathyroid Hormone Reduction after Total Thyroidectomy in Thyroid Cancer Patients

**DOI:** 10.1155/2021/8657918

**Published:** 2021-12-15

**Authors:** Yan Zhang, Weihui Zheng, Yuanyuan Huang, Chao Chen

**Affiliations:** ^1^Department of Head and Neck Surgery, The Cancer Hospital of the University of Chinese Academy of Sciences (Zhejiang Cancer Hospital), Institute of Basic Medicine and Cancer (IBMC), Chinese Academy of Sciences, No. 1 East Banshan Road, Hangzhou 310022, Zhejiang, China; ^2^Key Laboratory of Head and Neck Cancer Translational Research of Zhejiang Province, Hangzhou, China; ^3^Department of Surgery, Hangzhou Fuyang Women and Children Hospital, No. 25 Hengliangting Road, Hangzhou 311400, Zhejiang, China

## Abstract

**Objective:**

Given its role in the regulation of calcium and PTH levels, vitamin D was presumed as a potential predictor of postoperative hypoparathyroidism. However, the reports about their association were controversial. This study aims to reveal the relationship between preoperative vitamin D and postoperative parathyroid hormone (PTH).

**Methods:**

A total of 242 papillary thyroid cancer (PTC) patients who underwent total thyroidectomy (TT) during the period from June 2016 to December 2017 at our hospital were enrolled. Patients were divided into two groups, HypoP and Non-HypoP groups, based on postoperative PTH < 15.0 or ≥15.0 pg/mL, and ΔPTH_50_+ and ΔPTH_50_− groups, based on postoperative PTH reduction ratio ≥ 50% or <50%. Clinicopathological features and laboratory data were compared between two sets of groups.

**Results:**

Preoperative PTH level was lower in the HypoP group than in the Non-HypoP group (42.83 vs. 47.52 pg/mL, *p*=0.018). No significant difference of vitamin D insufficiency was found between the HypoP and Non-HypoP groups (80.8% vs. 74.1%, *p*=0.226). The rate of vitamin D insufficiency was higher in the ΔPTH_50_+ group than in the ΔPTH_50_− group (82.6% vs. 68.4%, *p*=0.010). By multivariate logistic regression analysis, vitamin D insufficiency was an independent predictor of postoperative PTH reduction ratio ≥ 50% (OR = 2.2, *p*=0.017).

**Conclusion:**

Vitamin D insufficiency is not associated with postoperative PTH in PTC patients undergoing TT. However, vitamin D insufficiency is an independent predictor of postoperative PTH reduction ratio.

## 1. Introduction

Globally, thyroid cancer was one of the cancers with the largest incidence increase in the recent decade for both sexes combined [1]. In China, thyroid cancer is the most commonly diagnosed cancer among young women [2]. Papillary thyroid cancer (PTC) is the most common pathological type of thyroid cancer, accounting for about 85% of thyroid cancer cases [3]. Total thyroidectomy (TT) is recommended for PTCs with tumors >4 cm in diameter or bilateral nodularity. One of the most common complications following TT is hypoparathyroidism. The incidence of transient postoperative hypoparathyroidism varies from 19% to 39%, while the rate of permanent hypoparathyroidism ranges from 0% to 3% [4]. Inadvertent resection and devascularization of the parathyroid glands are the most definite surgery-related risk factors of postoperative hypoparathyroidism. Besides, patient-related and disease-related risk factors, such as age, gender, vitamin D deficiency, and autoimmune thyroiditis, have been reported in previous studies [4–6].

Vitamin D plays a significant role in calcium homeostasis and metabolism. 25-hydroxyvitamin D (25OHD), as the circulating form of vitamin D in human blood, has an inverse relationship with parathyroid hormone (PTH) [7]. Vitamin D deficiency and insufficiency are global health problems. It is estimated that approximately 30% and 60% of children and adults worldwide are vitamin D deficient and insufficient, respectively [7]. Given its importance in calcium absorption and PTH interaction, some scholars assume that vitamin D deficiency is another potential risk factor of postoperative hypoparathyroidism. A number of studies have found a significant influence of vitamin D deficiency on postoperative hypoparathyroidism, while others did not observe this association [8–11].

This retrospective study aims to evaluate the potential effect of preoperative vitamin D level on postoperative hypoparathyroidism in a large consecutive group of patients who underwent TT for PTC in a tertiary cancer center.

## 2. Materials and Methods

### 2.1. Study Population

We retrospectively enrolled all consecutive adult patients who underwent TT for PTC at the Cancer Hospital of the University of Chinese Academy of Sciences between June 2016 and December 2017. All patients provided written informed consent prior to surgery. This study was reviewed and approved by the research ethics board at the Cancer Hospital of the University of the Chinese Academy of Sciences. Patients were excluded if they had concomitant diseases as follows: parathyroid hyperplasia, parathyroid adenoma, secondary hyperparathyroidism, preexisting thyroidectomy history, daily vitamin D supplementation, preexisting gastric bypass surgery, and corticosteroid medication. All patients followed the postoperative follow-up schedule and paid visits in our outpatient clinic 1, 3, and 6 months after surgery. Serum PTH and calcium levels were measured at each follow-up visit to evaluate the recovery process of postoperative hypoparathyroidism.

### 2.2. Surgical Technique

All surgeries were performed by one team of experienced oncology surgeons in our head and neck surgery department, using the conventional open TT technique by extracapsular dissection. Prophylactic ipsilateral central compartment neck dissection (CND) was performed concomitantly. Patients with bilateral foci underwent bilateral CND. Therapeutic lateral compartment neck dissection (LND) was performed in patients with preoperatively confirmed lateral lymph node metastasis. All parathyroid glands were meticulously preserved *in situ* with sufficient vasculature. Inadvertently removed or devascularized parathyroid glands were autotransplanted into the ipsilateral sternocleidomastoid muscle or strap muscle.

### 2.3. Laboratory Measurements

Preoperative serum 25OHD, intact PTH, total calcium, and albumin were measured in the morning following an 8-hour overnight fast within one month before surgery. Postoperative intact PTH, total calcium, and serum albumin were tested in the morning following surgery. Serum calcium was corrected for albumin concentration. Intravenous and/or oral calcium and calcitriol were not regularly administrated after surgery until postoperative blood test results were recommended or symptomatic hypocalcemia was presented. All blood tests were performed in our clinical laboratory department. The reference ranges of laboratory measurements were as follows: 2.00–2.60 mmol/L for serum calcium; 15.0–65.0 pg/mL for intact PTH; and ≥30–100 ng/mL for serum 25OHD.

### 2.4. Definitions

Biochemical hypoparathyroidism was defined as a serum intact PTH level <15.0 pg/mL. Hypocalcemia was defined as a serum calcium level <2.00 mmol/L with or without clinical hypocalcemic symptoms. Vitamin D insufficiency was defined as a serum 25OHD level <30 ng/mL. According to the American Thyroid Association (ATA) statement on postoperative hypoparathyroidism, transient hypoparathyroidism was defined as the condition that persisted for less than 6 months after surgery and permanent hypoparathyroidism for more than 6 months [12]. Postoperative PTH reduction ratio (∆PTH) was calculated as follows.

### 2.5. Outcome Assessment

Patients were divided into two groups based on postoperative PTH levels or ∆PTH values. Firstly, according to postoperative PTH levels, patients were classified into the postoperative hypoparathyroidism group (HypoP) and the nonhypoparathyroidism group (Non-HypoP). Secondly, according to ∆PTH values, patients were classified into the ∆PTH50+ group (∆PTH ≥ 50, postoperative PTH reduced 50% and more compared to preoperative PTH) and the ∆PTH50− group (∆PTH < 50, postoperative PTH reduced less than 50%). Groups in the two sets of classification were compared with respect to demographic, pathological characteristics, and laboratory measurements. Receiver operating characteristic (ROC) curves were performed to assess the diagnostic values of postoperative PTH and ∆PTH in predicting postoperative hypocalcemia.

### 2.6. Statistical Analysis

Continuous variables were presented as mean ± standard deviation (SD) for the normally distributed and median (interquartile range, IQR) for the nonnormally distributed. Categorical variables were presented as frequency (percentage). The normality of continuous variables was examined with nonparametric Kolmogorov–Smirnov test. Differences between the HypoP and Non-HypoP groups, as well as the ΔPTH_50_+ and ΔPTH_50_− groups, were tested using Student's *t*-test and Mann–Whitney *U* test for continuous variables and the Pearson Chi-square test for nominal variables. Multivariate logistic regression was performed to identify the predictive variables. All examinations were performed using IBM SPSS statistics (version 19.0). Two-sided *p* value < 0.05 was considered statistically significant for all tests.

## 3. Results

A total of 242 patients who underwent TT for PTC at our hospital were retrospectively enrolled in this study, including 190 females and 52 males. All patients had perioperative serum 25OHD, PTH, and calcium data except for 6 patients who missed either preoperative or postoperative calcium data. The median levels of preoperative and postoperative PTH were 44.04 (35.36–56.13) pg/mL and 19.13 (8.94–27.77) pg/mL, respectively (*p* < 0.001). The median level of postoperative PTH had a 56.6% reduction from preoperative PTH. Of all the patients, 99 (99/242, 40.9%) had transient postoperative hypoparathyroidism, but no one developed permanent hypoparathyroidism. The mean levels of preoperative and postoperative calcium were 2.351 ± 0.121 mmol/L and 2.071 ± 0.180 mmol/L, respectively (*p* < 0.001). 71 (71/242, 29.3%) had postoperative hypocalcemia, 26 (26/71, 36.6%) of whom showed hypocalcemic symptoms (perioral or distal acral paresthesia, tetany). 186 (186/242, 76.9%) patients suffered from vitamin D insufficiency.

Baseline demographic, pathological, and preoperative biochemical parameters of patients in the HypoP and Non-HypoP groups are shown in Table 1. The HypoP group had a lower preoperative PTH level than that of the Non-HypoP group (42.83 vs. 47.52, *p*=0.018). There was no difference in the two groups regarding sex, age, 25OHD, vitamin D insufficiency, preoperative calcium, extent of neck dissection, Hashimoto's thyroiditis, tumor diameter, and N stage (Table 1).

Baseline demographic, pathological, and preoperative biochemical parameters of patients in the ΔPTH_50_+ and ΔPTH_50_− groups are shown in Table 2. As compared to the ΔPTH_50_− group, the ΔPTH_50_+ group had a lower mean serum 25OHD level (24.1 vs. 26.4, *p*=0.020) and a higher proportion of vitamin D insufficient patients (82.6% vs. 68.4%, *p*=0.010). Besides, the ΔPTH_50_+ group had a higher proportion of female patients (83.3% vs. 71.4%, *p*=0.027) and a lower proportion of N1 stage patients (47.2% vs. 60.2%, *p*=0.047). No difference was found between the ΔPTH_50_+ and ΔPTH_50_− groups regarding age, preoperative calcium, extent of neck dissection, Hashimoto's thyroiditis, and tumor diameter (Table 2).

Multivariate analysis was performed comparing vitamin D insufficiency, gender, and N stage outcomes between the ΔPTH_50_+ and ΔPTH_50_− groups (Table 3). Only vitamin D insufficiency was significantly associated with the postoperative PTH reduction ratio (OR = 2.2, *p*=0.017).

Pre- and postoperative PTH and calcium levels were compared between vitamin D insufficient and sufficient patients in Table 4. Vitamin D insufficient patients had a higher median preoperative PTH level than vitamin D sufficient patients (46.15 vs. 39.59, *p*=0.038). No difference was found regarding preoperative calcium, postoperative PTH and calcium, hypoparathyroidism rate, and hypocalcemia rate (Table 4).

**Figure 1:**
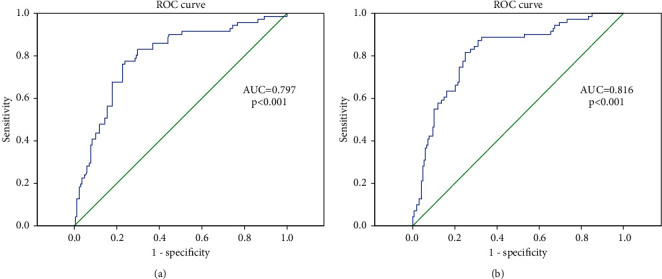
Receiver operating characteristic curve analysis of postoperative PTH parameters predicting postoperative hypocalcemia. (a) Postoperative PTH predicting postoperative hypocalcemia. (b) ΔPTH predicting postoperative hypocalcemia.

A ROC curve analysis was performed to evaluate the influence of postoperative PTH level and postoperative PTH reduction ratio on postoperative hypocalcemia (Figure 1). Both parameters were good predictors of postoperative hypocalcemia as the area under the curve (AUC) > 0.7. The best cutoff value for postoperative PTH was found to be 14.0 pg/mL with a sensitivity and specificity of 77.5% and 75.6%, respectively (AUC = 0.797, *p* < 0.001). The best cutoff value for ΔPTH was found to be 60% with a sensitivity and specificity of 87.3% and 69.6%, respectively (AUC = 0.816, *p* < 0.001).

## 4. Discussion

Postoperative hypoparathyroidism is the most common complication of total thyroidectomy at present. Despite the fact that postoperative hypoparathyroidism is asymptomatic and transient in most cases, it can lead to the consequences of extra blood tests, additional medications, and increased length of hospitalization. The incidence of transient and permanent hypoparathyroidism after TT varies from 5% to 60% and 0% to 12%, respectively, in the literature [13]. The wide variation of postoperative hypoparathyroidism incidence is caused partly by the discrepancies in the definition of hypoparathyroidism and the detection time of serum PTH [13]. In this study, the incidence of transient and permanent hypoparathyroidism was 29.3% and 0%, respectively.

Risk factors of postoperative hypoparathyroidism can be summarized as surgery-related, disease-related, and patient-related. The surgery-related etiologies included inadvertent resection and devascularization of the parathyroid glands. Advanced surgical skills and new identification techniques have improved the visual identification and viability of parathyroid glands [14]. In addition to surgical skills, the extent of neck dissection was presumed widely as another risk factor. Yoo et al. [15] and Raffaelli et al. [16] compared TT with ipsilateral CND versus TT with routine bilateral CND in the management of PTC. Both retrospective studies found bilateral CND was associated with a significantly increased rate of transient hypoparathyroidism. However, another retrospective study of 690 TT patients found a different result after controlling other confounding factors [17]. Huang et al. [17] showed the extent of CND was not independently associated with postoperative hypoparathyroidism after adjusting for other confounding factors such as surgeon, intraoperative carbon nanoparticle injection, and preoperative PTH. In our study, neither bilateral CND nor LND was statistically associated with postoperative hypoparathyroidism. We presumed that parathyroid gland preservation *in situ* and selective autotransplantation performed by experienced surgeons would minimize the effect of prophylactic and therapeutic neck dissection on postoperative hypoparathyroidism.

Disease-related and patient-related risk factors of postoperative hypoparathyroidism, such as age, gender, Graves' disease, autoimmune thyroiditis, and pathological characteristics, were reported in previous studies [5, 18, 19]. However, there was no concordant data. Some studies have identified older age as a risk factor while others have found younger age groups to be at risk [6]. In a meta-analysis of 115 observational studies of bilateral thyroid surgery, age was not significantly associated with postoperative transient hypocalcemia, while female gender and Graves' disease were independent risk factors [20]. Several studies have suggested that autoimmune thyroiditis and higher cancer stages could be related to a higher risk of postoperative hypoparathyroidism, but there are conflicting reports [4, 9, 11, 20]. In our study, gender, age, Hashimoto's thyroiditis, tumor size, and N stage were not significantly associated with postoperative hypoparathyroidism. However, in univariate analysis of the ΔPTH_50_− and ΔPTH_50_+ groups, female gender and N0 stage were statistically significant predictors of postoperative PTH reduction ratio ≥50% (*p* = 0.027 and 0.047, respectively), but not independent risk factors after adjusted with vitamin D insufficiency in multivariate analysis (*p* = 0.0282 and 0.104, respectively).

Vitamin D plays an important role in the regulation of calcium and PTH levels. Activated vitamin D increases serum calcium by increasing intestinal absorption and bone resorption while suppressing PTH secretion through increased serum calcium and vitamin D receptors in the parathyroid gland [7, 10]. Given its role in the regulation of calcium and PTH levels, vitamin D deficiency was presumed as a potential risk factor of postoperative hypocalcemia. The relationship of vitamin D and postoperative hypoparathyroidism or hypocalcemia has been evaluated in a number of studies, but the results are controversial. Several studies reported that low preoperative 25OHD was associated with postoperative hypoparathyroidism or hypocalcemia [11, 21–23]. Bove et al. [11] analyzed clinical and lab data of 177 patients who underwent total thyroidectomy and found preoperative vitamin D deficiency (<25 ng/mL) was an independent risk factor in the development of hypocalcemia (OR: 14.8, 95% CI: 1.59–59.70, *p* = 0.012). Recently, a meta-analysis was published by Vaitsi et al. [24] which included 39 observational studies of patients undergoing thyroidectomy with preoperative vitamin D status and postoperative hypoparathyroidism data. Patients with preoperative vitamin D deficiency demonstrated a higher risk for transient hypoparathyroidism compared with those with preoperative vitamin D sufficiency (RR: 1.92, 95% CI: 1.50–2.45, *I*^2^ = 85%) [24].

In contrast to the abovementioned studies, some studies found no significant association between preoperative 25OHD and postoperative hypoparathyroidism [9, 25–27]. Lin et al. [25] reported that there was no significant difference in the rates of biochemical or symptomatic hypocalcemia between the vitamin D insufficient group and the control group (*p*= 0.50 and *p* = 0.96, respectively) in a series of 152 patients undergoing near-total thyroidectomy. Cherian et al. [26] reported that mean postoperative PTH was not significantly different between the vitamin D deficient and sufficient groups (18.96 vs. 19.70, *p* = 0.71) in a series of 150 patients undergoing total thyroidectomy with or without neck dissection. Kim et al. [28] analyzed pre- and postoperative lab tests of 267 patients who underwent total thyroidectomy for thyroid cancer. In patients with preoperative vitamin D levels <10 ng/mL, the rate of biochemical hypocalcemia was significantly higher (74.0% vs. 60.3%, *p* = 0.045), but the rate of hypoparathyroidism (postoperative PTH < 15 pg/mL) showed no significant difference (54.8% vs. 56.2%, *p* = 0.89) [28].

The discordance of previous studies may be partly due to the heterogeneity of thyroid diseases or the relatively small subject population. Many studies enrolled patients with various thyroid diseases, such as retrosternal goiter, thyroid cancer, benign tumors, and hyperthyroidism. Unlike those studies, all patients in our study had PTC requiring TT and CND. In our study, vitamin D insufficiency was not significantly associated with hypoparathyroidism and hypocalcemia (*p* = 0.226 and 0.121, respectively). In all the preoperative and postoperative biochemical parameters, only preoperative PTH was significantly associated with vitamin D insufficiency (*p* = 0.038), which was in accordance with the inverse relationship of vitamin D and PTH. However, when postoperative PTH reduction ratio was adopted in the analysis, both 25OHD and vitamin D insufficiency rates were found significantly different between the groups of postoperative PTH reduction ratio ≥50% (ΔPTH_50_+) and the control group (ΔPTH_50_−). The ΔPTH_50_+ group had a lower mean 25OHD and a higher rate of vitamin D insufficiency (*p* = 0.020 and 0.010, respectively). In multivariate analysis, vitamin D insufficiency was significantly associated with the postoperative PTH reduction ratio ≥50% (OR = 2.2, *p* = 0.017).

Preoperative PTH was scarcely discussed in previous studies about its effect on postoperative hypoparathyroidism or hypocalcemia. In a few studies that did discuss its role, preoperative PTH had a positive correlation with postoperative PTH [17, 29]. Huang et al. [17] enrolled 690 PTC patients who underwent TT with or without CND to investigate the predictive factors for postoperative hypoparathyroidism (PTH < 15 pg/mL) on the first postoperative day. The preoperative PTH was significantly lower in the hypoparathyroidism group (42.94 vs. 48.5, *p* < 0.001). To further discuss its effect on the severity of postoperative hypoparathyroidism, Huang et al. [17] classified all patients in the hypoparathyroidism group into three subgroups: mild hypoparathyroidism (10 pg/mL ≤ PTH < 15 pg/mL), moderate (5 pg/mL ≤ PTH < 10 pg/mL), and severe (PTH < 5 pg/mL). The median preoperative PTH levels of these three subgroups were 45.1, 44.3, and 36.5 pg/mL, respectively (*p* < 0.001). This positive correlation of pre- and postoperative PTH was also verified in our study as the HypoP group showed a lower median preoperative PTH level (42.83 vs. 47.52, *p*=0.018).

To adjust the confounding effect of preoperative PTH on postoperative PTH, we adopted the postoperative PTH reduction ratio (ΔPTH) as the second measurement of postoperative PTH status. Kakava et al. [9] reported that both the first postoperative day PTH and ΔPTH were good predictors of hypocalcemia (AUC = 0.806, *p*=0.05 and AUC = 0.825, *p*=0.047, respectively). The ROC curve analysis indicated that the best cutoff for PTH was 9.4 pg/ml with a sensitivity of 84.9% and a specificity of 71.4% to predict hypocalcemia. And the best cutoff for ΔPTH was a 50% reduction from the preoperative PTH with a sensitivity of 76% and a specificity of 75% [9]. Same as the results in abovementioned study, our study also proved ΔPTH was a good predictor of postoperative hypocalcemia with a slightly higher value of AUC than postoperative PTH (AUC = 0.816, *p* < 0.001 vs. AUC = 0.797, *p* < 0.001).

## 5. Conclusions

Our study is among the first few to investigate risk factors of postoperative PTH reduction ratio. ΔPTH can control the confounding effect of preoperative PTH on postoperative PTH. Besides, ΔPTH is a good predictor of hypocalcemia, even slightly better than PTH. We found vitamin D insufficiency was independently associated with ΔPTH. Vitamin D insufficient patients were 2.2 times more likely to have a postoperative PTH reduction ratio of more than 50%. Therefore, preoperative supplementation of vitamin D is recommended as a way to minimize postoperative PTH reduction in PTC patients with vitamin D insufficiency. However, the retrospective nature and the moderate scale of the study population are the limitations of our study. The appropriate dose of vitamin D supplementation and optimal level of preoperative vitamin D level need to be evaluated in future prospective studies.

## Figures and Tables

**Table 1:** Demographic, pathological, and preoperative biochemical parameters of patients in the HypoP and Non-HypoP groups.

	HypoP (*N* = 99)	Non-HypoP (*N* = 143)	*p* value
Female, *n* (%)	82 (82.8)	108 (75.5)	0.174
Age (years), mean ± SD	47.4 ± 13.9	49.0 ± 12.9	0.340
25OHD (ng/mL), mean ± SD	24.5 ± 6.6	25.4 ± 7.9	0.333
Vitamin D insufficiency, *n* (%)	80 (80.8)	106 (74.1)	0.226
Preoperative PTH (pg/mL), median (IQR)	42.83 (32.85–50.62)	47.52 (36.67–59.98)	0.018
Preoperative calcium (mmol/L), mean ± SD	2.349 ± 0.110	2.350 ± 0.129	0.960
Bilateral CND, *n* (%)	54 (54.5)	89 (62.2)	0.231
LND, *n* (%)	30 (30.3)	40 (28.0)	0.694
Hashimoto's thyroiditis, *n* (%)	13 (13.1)	22 (15.4)	0.624
Tumor > 1 cm, *n* (%)	46 (46.5)	71 (49.7)	0.626
N1 stage, *n* (%)	47 (47.5)	80 (55.9)	0.195

25OHD, 25-hydroxyvitamin D; PTH, parathyroid hormone; CCND, central compartment neck dissection; LCND, lateral compartment neck dissection.

**Table 2:** Demographic, pathological, and preoperative biochemical parameters of patients in the ΔPTH_50_+ and ΔPTH_50_− groups.

	ΔPTH_50_+ (*N* = 144)	ΔPTH_50_−*N* = 98)	*p* value
Female, *n* (%)	120 (83.3)	70 (71.4)	0.027
Age (years), mean ± SD	47.0 ± 13.5	50.2 ± 12.8	0.069
25OHD (ng/mL), mean ± SD	24.1 ± 6.6	26.4 ± 8.1	0.020
Vitamin D insufficiency, *n* (%)	119 (82.6)	67 (68.4)	0.010
Preoperative calcium (mmol/L), mean ± SD	2.346 ± 0.112	2.354 ± 0.135	0.609
Bilateral CND, *n* (%)	81 (56.3)	62 (63.3)	0.276
LND, *n* (%)	40 (27.8)	30 (30.6)	0.633
Hashimoto's thyroiditis, *n* (%)	23 (16.0)	12 (12.2)	0.418
Tumor > 1 cm, *n* (%)	69 (47.9)	48 (49.0)	0.871
N1 stage, *n* (%)	68 (47.2)	59 (60.2)	0.047

25OHD, 25-hydroxyvitamin D; CCND, central compartment neck dissection; LCND, lateral compartment neck dissection.

**Table 3:** Multivariate logistic regression analysis in the ΔPTH_50_+ and ΔPTH_50_− groups.

Risk factor	*β*	*p* value	OR (95% CI)
Vitamin D insufficiency	0.767	0.017	2.153(1.147–4.040)
Female	0.373	0.282	1.452(0.737–2.861)
N1 stage	−0.470	0.104	0.625(0.355–1.101)

**Table 4:** Biochemical parameters of vitamin D insufficient and sufficient patients.

Biochemical parameters	Vitamin D insufficiency (*N* = 186)	Vitamin D sufficiency (*N* = 56)	*p* value
Preoperative PTH (pg/mL), median (IQR)	46.15 (35.84–57.44)	39.59 (33.04–51.90)	0.038
Preoperative calcium (mmol/L), mean ± SD	2.343 ± 0.118	2.371 ± 0.132	0.125
Postoperative PTH (pg/mL), median (IQR)	19.06 (8.60–27.42)	19.55 (10.90–30.56)	0.293
Postoperative calcium (mmol/L), mean ± SD	2.062 ± 0.177	2.095 ± 0.190	0.271
Hypoparathyroidism, *n* (%)	80 (43.0)	19 (33.9)	0.226
Hypocalcemia, *n* (%)	59 (31.7)	12 (21.4)	0.121

## Data Availability

The data used to support the findings of this study are available from the corresponding author upon request.
